# The role of the broader autism phenotype in anxiety and depression in college-aged adults

**DOI:** 10.3389/fpsyt.2023.1187298

**Published:** 2023-06-05

**Authors:** McKayla R. Kurtz, Rajesh K. Kana, Daphne L. Rivera, Sharlene D. Newman

**Affiliations:** ^1^Department of Psychology, Center for Innovative Research in Autism, The University of Alabama, Tuscaloosa, AL, United States; ^2^Department of Psychological and Brain Sciences, Indiana University, Bloomington, IN, United States; ^3^Alabama Life Research Institute, The University of Alabama, Tuscaloosa, AL, United States

**Keywords:** autistic traits, anxiety, depression, race, socioeconomic status

## Abstract

The current study examines the relationship between the presence of autistic traits and anxiety and mood disorders in young adults from different racial groups. A representative sample from a predominately white university (2,791 non-Hispanic White (NHW) and 185 Black students) completed the broad autism phenotype questionnaire (BAPQ), a measure of depression (Patient Health Questionnaire, PHQ-9), and anxiety (Generalized Anxiety Disorder, GAD-7). Statistical Package for Social Sciences (SPSS) was used to perform two multiple regression analyses to determine the association between race, BAPQ score and anxiety and depression symptoms. The current study found a stronger association between autistic traits had depression and anxiety symptoms in Black participants than did NHW participants. These findings underscore the association between autistic traits and anxiety and depression in Black communities, and the need for further studies on this topic area. Additionally, it highlights the importance of improving access to mental health care for this population.

## Introduction

Prior to 2020, mental health disorders were included as one of the top 10 leading causes of burden worldwide, with depression being the fourth leading cause (1). Mental health disorders, specifically anxiety and depression, are often associated with decreased quality of life (2, 3) and increased mortality rates (4). Additionally, the prevalence of mood and anxiety disorders in the general population is high. A study based on data collected in 2012 through 2013 found that among a population of 36,309 US adults, approximately 10.4% experienced a 12-month Major Depressive Disorder and 20.6% experienced a lifetime Major Depressive Disorder (5). (6) Reported that 33.7% of the general population experience an anxiety disorder during their lifetime. Several studies have also shown that the overall rate of mood disorders in the general population has increased over the past decade. One study found that there was a 63% increase in the number of individuals aged 18–25 years who reported experiencing a major depressive episode between 2009 and 2017 (7). Similarly, among undergraduates specifically, the number of students who experienced severe depression, engaged in self-harm, and attempted or planned suicide doubled between 2012 and 2018. Additionally, rates of moderate to severe anxiety increased by approximately 92% (8). These findings emphasize the importance of studying the underlying mechanisms responsible for the increase in anxiety and mood disorders among adult and college age populations as well as developing better mental health care and more effective treatment and intervention plans.

Internalizing problems – increased depression and anxiety symptoms – are also very prominent in individuals with autism spectrum disorder (ASD) as well as those with broader autistic phenotypic (BAP) traits. ASD is a neurodevelopmental condition characterized by restricted and repetitive behaviors and interests, and difficulties with social communication and interactions (9). Research suggests relatives of autistic individuals often display autistic tendencies but do not experience the same functional impairment as those with the condition. Therefore, these individuals do not meet criteria for a clinical diagnosis. The sub-diagnostic autistic traits are referred to as BAP (10). BAP traits include pragmatic difficulties, broadly defined stereotyped behaviors and communication difficulties, social skill and emotion recognition differences, rigidity, and aloofness (11–13). While the BAP is often seen in relatives of autistic individuals, they are also present amongst the general population without autistic relatives (14–16). However, less research has examined the relationship between mood and anxiety symptoms and those with sub-diagnostic autism traits. The BAP is associated with increased rates of psychological disorders and difficulties in a variety of cognitive domains. For example, individuals with the BAP often experience difficulties with cognitive functions such as central coherence, executive function, and neurological processing in general (17). Additionally, (18) reported higher rates of obsessive–compulsive disorder in relatives of autistic individuals. Research has also shown a relationship between anxiety and mood disorders and those with the BAP. For example, those with the more autistic traits are shown to exhibit more symptoms of depression and anxiety than those with less autistic traits (19–22). Furthermore, adults who reported a history of depression reported more autistic traits than those without a history of depression (23). Because depression is often linked to suicidal ideation and suicide attempts, it is important to note that studies have also found an increased risk of suicide in individuals with autistic traits (24). For example, through a survey that targeted suicide prevention websites and social media, Richards et al. (25) found that autistic traits, as measured by the Autism Spectrum Quotient (AQ), were higher in those who had attempted suicide.

The prevalence of mood and anxiety disorders among Black populations compared to Non-Hispanic White populations has been reported to be lower, although the chronicity and severity are typically higher. In the Twenge et al. (7) study that reported increases in those who had experienced a major depressive episode, the largest increase came from White Americans, followed by Hispanic and Black Americans, respectively. A study by Hasin et al. (5) examined prevalence rates among 36,309 American adults in 2012 and 2013 and reported the odds of a 12-month Major Depressive Disorder were lower among African American, Asian, and Hispanic adults compared to Non-Hispanic White adults. Despite the lower prevalence, the persistence of these disorders is often higher in Black populations (26, 27). One study found that, out of a group of Black males diagnosed with a mood disorder, only half of them made contact with a medical or mental health provider regarding their mental health condition. A large portion of these males with a mood or anxiety disorder had one that could be classified as lifetime or chronic (28). A similar study found that 12-month prevalence of mood and anxiety disorders were lower among Non-Hispanic Black participants compared to Non-Hispanic White participants, but that the overall persistence of these disorders was greater in the Non-Hispanic Black population (29). Recent data have also reported that Black adolescents had an increase in the rate of suicide attempts (30, 31). This increase was observed even though the rate in non-Hispanic White (NHW) adolescents was unchanged (32).

The previous work examining increased suicide attempts in both autistic and Black communities, while not the main topic of the current work, motivated the current study. The goal of the current, exploratory study was to lay a foundation for further research examining the differential outcomes and experiences of Black individuals with autistic traits. Specifically, in relation to mood and anxiety symptoms in this population. Black individuals experience high levels of social stress, including racial stress (33). This increased level of stress is expected to contribute to increases in frequency of anxiety and mood symptoms and is expected to interact with the presence of autistic traits to result in poorer outcomes. The current study aims to examine the prevalence of autistic traits and their relationship to depression and anxiety symptoms in Black and non-Hispanic white populations, in which autistic traits were measured using the Broad Autism Phenotype Questionnaire (BAPQ) in a sample of college students from a large Midwestern university.

## Method

### Participants

This sample comprised of 2976 College-age adults (903 male; 2073; female). Their ages ranged from 17 to 46 years (*M* = 19.06, SD = 1.658). Participants completed a series of surveys and questionnaires as part of an introductory psychology course at Midwestern University. The study was conducted in a span of 18 months (between 2015 and 2017). Informed consent was obtained from all participants prior to their participation. The research protocol was approved by the Institutional Review Board for Human subjects research at the Midwestern university. Participants who did not complete the BAPQ were excluded. Additionally, because of the focus of the study, only participants who identified as non-Hispanic White (NHW; *N* = 2791; 73% of original sample) and Black/African American (*N* = 185; 4.8% of original sample) were included in the analyses (see Table 1 for summary); this distribution of race matches the university’s demographics. The sample included seven individuals with a reported diagnosis of ASD (all NWH). Additionally, although the sample was limited to undergraduates, 20 were over the age of 25 (0.67% of the sample).

**Table 1:** Demographic information by group.

Demographic information by group (*n* = 2,976)								
Variable	Black (*n* = 185)			NHW (*n* = 2,791)			*T*-test comparing Black and NHW	
	*M*	SD	Range	*M*	SD	Range	*t*	*p*
Age (years)	19.3	1.31	17–25	19.04	1.68	17–46	−2.03	0.47
Annual family income	3.0 ($50–100k)	1.71		4.0 ($100–150k)	1.27		–	–
Parental education	3.7 (some college)	1.49		3.6 (some college)	0.98		–	–
BAPQ	3.4	0.53	0.0–4.1	3.37	0.56	0.0–4.4	−0.847	0.02*
PHQ9	1.69	2.28	0–8	1.29	1.98	0–8	−2.604	<0.001***
GAD7	0.95	1.56	0–6	0.7	1.31	0–6	−2.409	0.002**
	*n* (%)			*n* (%)				
Male	47 (25.4)			856 (30.7)				
Female	138 (74.6)			1935 (69.3)				
Family history depression	15 (8.1)			541 (19.4)				
Family history anxiety	5 (2.7)			192 (6.9)				
Family history autism	1 (0.5)			88 (3.2)				

BAPQ, Broad Autism Phenotype Questionnaire; PHQ9, Patient Health Questionnaire; GAD7, General Anxiety Disorder; NHW, Non-Hispanic White. *p < 0.05, **p < 0.01, ***p < 0.001.

### Measures

**Figure 1:**
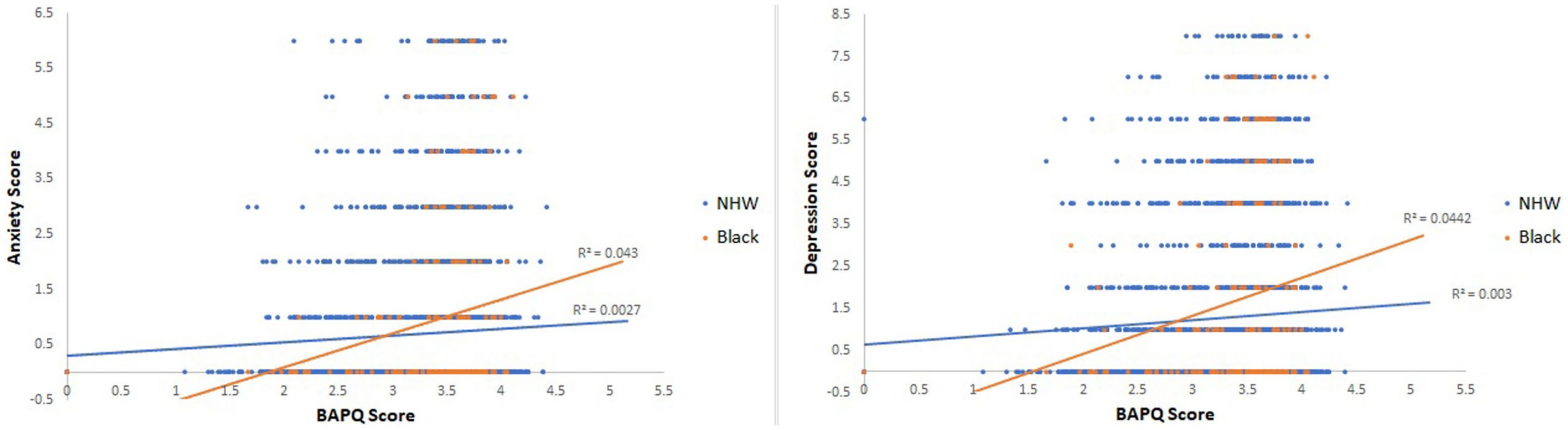
Scatterplots depicting the interaction between race and BAPQ for anxiety and depression symptoms.

A family history survey was administered to obtain a family history of anxiety, depression and autism [responses were binary (yes/no, e.g., “Has your parent or grandparent been diagnosed with ____?”), annual family income (multiple choice: (1) under $25k, (2) $25–50k, (3) $50–100k, (4) $100–150k, or (5) over $150k) and the highest level of parental education (multiple choice: (1) did not complete high school, (2) high school, (3) some college, (4) no degree, college graduate (4 or 2 year), or (5) professional/graduate school)]. Participants’ race, gender, and age were also obtained (Figure 1).

### Broad autism phenotype questionnaire

The Broader Autism Phenotype (BAP) is a set of autistic traits in individuals who do not necessarily have an autism diagnosis, particularly relatives of autistic individuals (34). Therefore, the BAPQ can be utilized in assessing the overall autistic traits of a population when obtaining clinical assessment for all participants is not possible (34, 35). The BAPQ is a 36-item assessment divided among three subscales: *social behavior, stereotyped-repetitive behavior, and communication*, with a six-point scale for responding to each question. For example, one of the items says, “I find it hard to get my words out smoothly” (34). The overall score ranges from 1 to 6 and is averaged from all 36 questions. A cut-off of 3.13 was used to label high and low scores based on the cutoff previously reported by Hurley and colleagues (2006), which resulted in 715 low and 2261 high-scores (see Supplementary Data for an analysis using a higher cutoff of 3.5).

### Patient Health Questionnaire (PHQ-9)

Depressive symptoms were assessed using the PHQ-9 (36–39). Each of the nine PHQ-9 depression items describes one symptom corresponding to one of the nine diagnostics in the Diagnostic and Statistical Manual of Mental Disorders, fourth edition (DSM-4). Participants are asked to rank how often in the last 2 weeks they have experienced that symptom from “Not at all” to “Nearly every day.” For example, one of the items is “Little interest or pleasure in doing things” (38). The PHQ-9 is a valid measure of depression severity due to its correlation with the DSM-4 requirements for a depression diagnosis and is useful when gathering a clinical diagnosis is not feasible (36).

### General Anxiety Disorder-7 (GAD-7)

Anxiety symptoms were assessed using the GAD-7, which consists of 7 items asking participants to rate how frequently in the last 2 weeks they have experienced a specific anxiety symptom from “Not at all” to “Nearly every day” (37). For example, one of the items is “Not being able to stop or control worry.” Scores can range from 0 to 27, with 5 representing mild, 10 representing moderate, and 15 representing severe anxiety symptoms. The GAD-7 has been found to be a valid measure of generalized anxiety symptoms based on DSM-4 criteria and is useful when a clinical diagnosis is not feasible (37).

### Statistical analyses

Statistical Package for Social Sciences (SPSS) for Windows, version 28 was used for statistical analysis. Descriptive statistics were computed to evaluate participant characteristics (i.e., mean, standard deviation, and frequencies). Additionally, multiple regression analyses were performed to determine the association between race (Black and NHW), BAPQ scores, and anxiety and depression symptoms (An ANOVA is presented in the Supplementary materials).

## Results

A multiple regression analysis was conducted to examine the relationship between anxiety scores, autistic traits, and race. The results revealed a main effect of BAPQ scores (*b* = 0.147, *t*(2973) = 3.4, *p* < 0.001) and race (*b* = 0.24, *t*(2973) = 2.4, *p* = 0.02). These main effects indicate that, on average, Black participants and participants with more autistic traits experienced more anxiety symptoms. However, there was a significant interaction between BAPQ scores and anxiety symptoms (*b* = 0.49, *t*(2972) = 2.57, *p* = 0.01). Specifically, the slope relating BAPQ to anxiety scores was *b* = 0.12, *t*(2972) = 2.71, *p* = 0.0068 in NHW participants and *b* = 0.61, *t*(2972) = 3.31, *p* < 0.001 in Black participants. While both groups showed a significant effect of BAPQ on anxiety scores, the significant interaction indicates that the relationship was stronger for Black participants (Table 2).

**Table 2:** Summary of multiple regression analysis for variables predicting anxiety scores.

Variable	*B*	*t*	*Df*	*p*
BAPQ (main effect)	0.15	3.4	2973	<0.001***
Race (main effect)	0.24	2.4	2973	0.02*
BAPQ*Race	0.49	2.57	2972	0.01**
BAPQ at NHW	0.12	2.71	2972	0.01**
BAPQ at Black	0.61	3.31	2972	<0.001***

BAPQ, Broad Autism Phenotype Questionnaire; NHW, Non-Hispanic White. *p < 0.05, **p < 0.01, ***p < 0.001.

A multiple regression analysis was conducted to examine the relationship between depression scores, autistic traits, and race. The results revealed a main effect of BAPQ scores (*b* = 0.23, *t*(2973) = 3.53, *p* < 0.001) and race (*b* = 0.39, *t*(2973) = 2.55, *p* = 0.01). These main effects indicate that, on average, Black participants and participants with more autistic traits experienced more depression symptoms. However, there was a significant interaction between BAPQ scores and depression symptoms (*b* = 0.72, *t*(2972) = 2.50, *p* = 0.01). Specifically, the slope relating BAPQ to depression scores was *b* = 0.19, *t*(2972) = 2.85, *p* = 0.004 in NHW participants and *b* = 0.91, *t*(2972) = 3.25, *p* = 0.001 in Black participants. While both groups showed a significant effect of BAPQ on depression scores, the significant interaction indicates that the relationship was stronger for Black participants (Table 3).

**Table 3:** Summary of multiple regression analysis for variables predicting depression scores.

Variable	*B*	*t*	*Df*	*p*
BAPQ (main effect)	0.23	3.53	2973	<0.001***
Race (main effect)	0.39	2.55	2973	0.01**
BAPQ*Race	0.72	2.50	2972	0.01**
BAPQ at NHW	0.19	2.85	2972	0.004**
BAPQ at Black	0.91	3.25	2972	0.001**

BAPQ, Broad Autism Phenotype Questionnaire; NHW, Non-Hispanic White. **p < 0.01, ***p < 0.001.

## Discussion

The goal of the present study was to examine whether autistic traits differentially affect Black undergraduate college students. The results revealed that although BAPQ scores were not different between Black and NHW participants (see Supplementary materials), there was a significant difference in the relationship between anxiety and mood symptoms and BAPQ. The current study found an interaction between race and BAPQ scores, suggesting that Black individuals with more BAP traits may be at a higher risk for experiencing symptoms of depression and anxiety than NHW individuals with BAP traits. These findings are significant given the already increased rates of anxiety and mood disorders (40–42) and attempted suicide (25, 43–45) in autistic individuals. This, coupled with prior findings of an increase in suicide attempts in Black adolescents (32) suggests higher rates of mood disorders and suicide attempts may be even greater among Black individuals with BAP traits. This is surprising considering consistent previous findings of a lower rate of psychological disorders, including depression, in Black compared to NHW populations [see (33) for review]. This is despite findings that Black populations have higher levels of psychosocial stress (46), which is positively correlated with psychological disorders.

It is important to consider the possible role of stress, particularly race-related stressors in this sample. Previous studies have reported that individuals with ASD and BAP have higher levels of perceived stress and lower perception of coping ability (47). This would suggest that the consequences of stress, including increased internalizing symptoms would be greater for those with higher BAP traits. Studies indicate Black individuals frequently encounter negative racial experiences, such as microaggressions from peers and faculty at primarily white institutions (PWIs) (48). Furthermore, research has shown that race-related stressors have a negative impact on Black individuals’ mental health and psychological well-being (49, 50). For instance, Black individuals who pursue psychotherapy, listed race-related stressors as a problem that led them to pursue psychotherapy (51). In addition, (52) found that psychological well-being was negatively correlated with autistic traits. Given the current study was conducted at a PWI, it is possible the interplay between autistic traits and race-related stressors are impacting the likelihood that Black individuals with autistic traits are at a greater risk for experiencing anxiety and mood disorders.

### Limitations

One primary limitation of this study is that the sample utilized is not fully representative of a larger population; it is composed primarily of white, female undergraduates from a Midwestern university. Additionally, while representative of the population from which it was drawn, the sample contains a much larger number of NHW participants than Black participants, making it difficult to draw definitive conclusions related to race.

### Implications

The current exploratory study is a first step in developing an understanding of the underlying mechanisms responsible for the racial disparities in mental health care and the relationship between autistic traits and anxiety and mood disorders, specifically in Black populations. Overall, these findings suggest BAP traits may exacerbate anxiety and mood symptoms in Black populations and highlight the importance of thoroughly assessing the relationship between these disorders and individuals with BAP traits. The results of the current study have also precipitated a few additional questions that are worthy of further exploration. For example, the participants in this study were all college students at a PWI in a predominately White college town, likely making the environment itself more stressful for many Black participants. However, it is unclear how this stress may interact with the presence of BAP traits. Future studies should examine how race-related stress may impact those with BAP traits. There is also the question of how socioeconomic status may interact with the relationship between BAP traits and anxiety and mood symptoms. Socioeconomic factors include both educational attainment as well as income and while parental educational attainment was similar across race, income was different which may contribute to racial stress. Future research should explore the role of this complex SES finding and how race may interact with it to affect mood disorders and BAP traits in larger, and more diverse samples.

## Data availability statement

The raw data supporting the conclusions of this article will be made available by the authors, without undue reservation.

## Ethics statement

The studies involving human participants were reviewed and approved by the Indiana University IRB. The patients/participants provided their written informed consent to participate in this study.

## Author contributions

SN was responsible for the design of the study, data collection, directing the analysis, interpretation, and writing of the manuscript. MK was involved in data analysis and writing. RK contributed to interpretation of results. DR contributed to data analysis. All authors contributed to the article and approved the submitted version.

## Funding

This project was funded by the Indiana Clinical and Translational Sciences Institute, funded in part by grant # UL1TR002529 from the National Institutes of Health, National Center for Advancing Translational Sciences, Clinical and Translational Sciences Award. The content is solely the responsibility of the authors and does not necessarily represent the official views of the National Institutes of Health.

## Conflict of interest

The authors declare that the research was conducted in the absence of any commercial or financial relationships that could be construed as a potential conflict of interest.

## Publisher’s note

All claims expressed in this article are solely those of the authors and do not necessarily represent those of their affiliated organizations, or those of the publisher, the editors and the reviewers. Any product that may be evaluated in this article, or claim that may be made by its manufacturer, is not guaranteed or endorsed by the publisher.
