# Supplementary material for: The role of the broader autism phenotype in anxiety and depression in college-aged adults
Source: Front Psychiatry. 2023 Jun 5;14:1187298. doi: 10.3389/fpsyt.2023.1187298 (PMC10278885; doi:10.3389/fpsyt.2023.1187298)

Supplementary Materials

A 2 (race: Black, non-Hispanic White) x 2 (BAPQ: high, low) between-subjects ANOVA using anxiety scores did not reveal a significant main effect for race (F(1,2976)=.36, *p*=.547, partial η2 = .000). However, this analysis showed a significant main effect for BAPQ scores (*F*(1,2976)= 9.77, *p*=.002, partial η2 = .003). Lastly, there was an interaction between race and BAPQ scores (*F*(1,2976)= 4.29, *p*=.038, partial η2 = .001). There were no significant differences in anxiety scores between Black and NHW participants who scored low on the BAPQ (*F*(1,2972)= .672, *p*=.412, partial η2 = .000). However, there were significant differences in anxiety scores between Black and NHW participants who scored high on the BAPQ (*F*(1,2972)= 9.072, *p*=.003, partial η2 = .003), such that Black participants had higher anxiety scores (M=1.074, SD=.108) than did NHW participants (M=.736, SD=.029) (see Table S1, Figure F1).

Table S1: Factorial ANOVA of BAPQ and Race on Anxiety scores

2X2 Factorial ANOVA of BAPQ and Race on Anxiety scores				
**Variable**	***F***	***df***	***p***	***partial η^2***
(Intercept)	125.27	1	<.001***	0.040
BAPQ	9.79	1	0.002**	0.003
Race	0.36	1	0.547	0.000
BAPQ*Race	4.29	1	0.038*	0.001
Note: *p<.05. **p<.01. ***p<.001				

A 2 (race: Black, NHW) x 2 (BAPQ: high, low) between-subjects ANOVA using depression scores did not reveal a significant main effect of race (F(1,2976)=.31, *p*=.577, partial η2 = .000). However, it revealed a significant main effect for BAPQ scores (*F*(1,2976)= 13.84, *p*<.001, partial η2 = .005). Lastly, there was an interaction between race and BAPQ scores (*F*(1,2976)= 5.66, *p*=.017, partial η2 = .002). There were no significant differences in depression scores between Black and NHW participants who

scored low on the BAPQ (F(1,2972)= 1.033, p=.310, partial η2 = .000). However, there were significant differences in depression scores between Black and NHW participants who scored high on the BAPQ (F(1,2972)= 10.939, p<.001, partial η2 = .004), such that Black participants had higher depression scores (M=1.913, SD=.164) than did NHW participants (M=1.353, SD=.043) (see Table S2 about here).

Table S2: Factorial ANOVA of BAPQ and Race on Depression scores

2X2 Factorial ANOVA of BAPQ and Race on Depression scores				
**Variable**	***F***	***df***	***p***	***partial*** η^2
(Intercept)	179.80	1	<.001***	0.057
BAPQ	13.84	1	<.001***	0.005
Race	0.31	1	0.577	0.000
BAPQ*Race	5.66	1	0.017*	0.002
Note: *p<.05. **p<.01. ***p<.001				

Figure 1: Depression and anxiety scores by race and BAPQ.

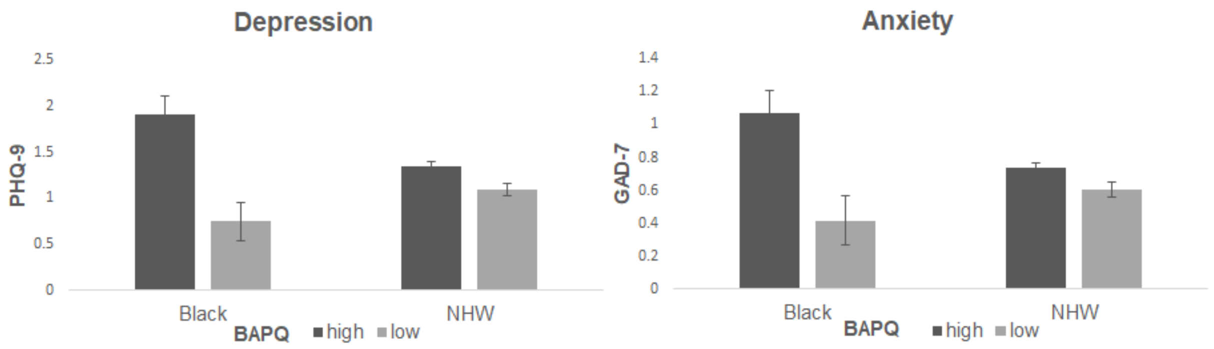

Supplement: Supplementary file 1 [file Data_Sheet_1.PDF]
